# Capgras syndrome conceptualization: from a delusional disorder to a structural neurological phenomenon

**DOI:** 10.1192/j.eurpsy.2023.2278

**Published:** 2023-07-19

**Authors:** M. M. Marques, L. Lopes, I. Grenha, J. Reis, F. Araujo, T. Novo

**Affiliations:** Psychiatry, Unidade Local de Saúde Alto Minho, Viana do Castelo, Portugal

## Abstract

**Introduction:**

The Capgras syndrome, also known as the delusion of doubles, is a delusional misidentification syndrome, defined in 1923 by Joseph Capgras, who referred to it as “*l’illusion des sosies*”, which means “the illusion of look-alikes”. In this syndrome, people falsely believe that someone significant to them has been replaced by an identical-looking impostor.

**Objectives:**

To review the evolution of the conceptualization of Capgras syndrome and its relationship with neurological disorders, such as dementia.

**Methods:**

Non-systematic review of the literature with selection of scientific articles published in the last 10 years, using *PUBMED* as database and the following keywords: «Capgras syndrome» and «dementia». 11 studies were included.

**Results:**

Originally, Capgras syndrome was seen exclusively as a psychiatric disorder: a delusional disorder, which can be associated to schizophrenia, bipolar or schizoaffective disorder. Since 1980, when organic brain lesions were identified in patients with Capgras syndrome, it started to be understood as a neuropsychiatric disorder. Previous studies revealed that in Capgras syndrome there is damage in the bifrontal, temporal cortex and the limbic system, structures that are involved in emotional arousal to familiar faces. In fact, Capgras Syndrome can be experienced in neurological conditions, including Alzheimer’s disease, dementia with Lewy body, Parkinson’s disease, epilepsy, cerebrovascular disease, subarachnoid hemorrhage, pituitary tumors and head injury. A 2014’s study showed that 73% of Capgras syndrome cases had comorbid diagnosis of schizophrenia, 26,4% had dementia and 16,7% had mood disorders. The prevalence of Capgras syndrome in neurodegenerative disorders is well known, and it is higher in dementia with Lewy body than in Alzheimer’s disease and frontotemporal dementia. In patients without a neurodegenerative disease, Capgras syndrome typically occurs at a younger age and is associated with psychiatric disease, cerebrovascular events, or illicit drug use. To date, it is unclear whether there are differences between Capgras syndrome as it occurs in neurodegenerative compared with non-neurodegenerative diseases.

**Conclusions:**

Currently, it is believed that Capgras syndrome can be associated not only with psychiatric diseases (a delusional syndrome, when belief evaluation is affected) but also with neurological diseases, such as neurodegenerative disorders. Therefore, when addressing a Capgras syndrome it is necessary to rule out these neurological conditions. Also, correct early identification of the Capgras syndrome in dementia cases will improve the clinical management, outcome and quality of life of patients and caregivers.

**Disclosure of Interest:**

None Declared

